# *Borrelia burgdorferi* Sensu Lato in Questing and Engorged Ticks from Different Habitat Types in Southern Germany

**DOI:** 10.3390/microorganisms9061266

**Published:** 2021-06-10

**Authors:** Cristian Răileanu, Cornelia Silaghi, Volker Fingerle, Gabriele Margos, Claudia Thiel, Kurt Pfister, Evelyn Overzier

**Affiliations:** 1 Institute of Infectology, Friedrich-Loeffler-Institut, Federal Research Institute for Animal Health, Südufer 10, 17493 Greifswald-Insel Riems, Germany; cristian.raileanu@fli.de; 2 Comparative Tropical Medicine and Parasitology, Ludwig-Maximilians-Universität München, 80805 Munich, Germany; claudia.thiel@med.uni-muenchen.de (C.T.); kurt.pfister@para.vetmed.uni-muenchen.de (K.P.); evelyn.overzier@gmx.de (E.O.); 3 National Reference Center for Borrelia, Bavarian Health and Food Safety Authority (LGL), 85764 Oberschleißheim, Germany; volker.fingerle@lgl.bayern.de (V.F.); gabriele.margos@lgl.bayern.de (G.M.)

**Keywords:** *Borrelia burgdorferi* sensu lato, *Ixodes ricinus*, epidemiology, urban area, pasture, forest, roe deer, wild boar, cattle, Germany

## Abstract

*Borrelia burgdorferi* sensu lato (s.l.) causes the most common tick-borne infection in Europe, with Germany being amongst the countries with the highest incidences in humans. This study aimed at (1) comparing infection rates of *B. burgdorferi* s.l. in questing *Ixodes ricinus* ticks from different habitat types in Southern Germany, (2) analysing genospecies distribution by habitat type, and (3) testing tissue and ticks from hosts for *B. burgdorferi* s.l. Questing ticks from urban, pasture, and natural habitats together with feeding ticks from cattle (pasture) and ticks and tissue samples from wild boars and roe deer (natural site) were tested by PCR and RFLP for species differentiation. *B. burgdorferi* s.l. was found in 29.8% questing adults and 15% nymphs. Prevalence was lower at the urban sites with occurrence of roe deer than where these were absent. *Borrelia burgdorferi* s.l. DNA was found in 4.8% ticks from roe deer, 6.3% from wild boar, and 7.8% from cattle. Six genospecies were identified in unfed ticks: *Borrelia afzelii* (48.6%), *Borrelia burgdorferi* sensu stricto (16%), *Borrelia garinii* (13.2%)*, Borrelia valaisiana* (7.5%), *Borrelia spielmanii* (6.2%), and *Borrelia bavariensis* (0.9%). This study shows high infection levels and a great diversity of *Borrelia* in questing ticks. The presence of roe deer seems to reduce *B. burgdorferi* s.l. infection rates in tick populations.

## 1. Introduction

Spirochetes of the *Borrelia burgdorferi* sensu lato (s.l.) complex cause Lyme borreliosis, the most common tick-borne infection in humans in Europe [1,2]. The annual estimated number of human cases on the continent is 64,500, with Germany being one of the countries with the highest incidence rates alongside Austria, Slovenia, and Sweden [3,4]. At least five of the 22 identified genospecies are known to be pathogenic to humans, causing a large spectrum of clinical manifestations [1,5,6,7]. Of these human pathogenic genospecies, *B. afzelii* and *B. garinii* are the most common ones in Europe [8]. The main tick vector for *B. burgdorferi* s.l. in Europe is *Ixodes ricinus* [9].

The geographic distribution and abundance of *B. burgdorferi* s.l. are influenced by several factors including the presence of the competent reservoir host species and the presence and density of the vector tick species, which in turn are influenced by the type of habitats.

Regarding habitat types, it has been previously described that *B. burgdorferi* s.l. is more commonly found in woodlands than in other types of habitat [9,10]. It was suggested that in woodlands, more favourable environmental conditions are found, allowing the expansion of tick populations and, combined with high densities of competent reservoir hosts (rodents, birds, and shrews), facilitating the abundance of *B. burgdorferi* genospecies [11]. Additionally, forest fragmentation and a low-grade biodiversity (e.g., low abundance of large mammals that are incompetent reservoir hosts) contribute greatly to the emergence of Lyme disease [11,12,13]. Pastures and urban and peri-urban ecotones located near forests can also sustain high abundance of *B. burgdorferi* s.l. species. Urban and peri-urban areas, in particular, can represent high-risk zones for public health due to human behaviour (i.e., the frequency of visits to these areas for recreational purposes). In addition to the increased human–tick contact, the risk exists also due to the dominance of competent reservoir hosts that correlates with the low presence or absence of non-competent reservoir animals and the existence of host species-poor communities (no dilution effect) [11,14,15].

Different hosts serve as reservoirs for different *B. burgdorferi* genospecies. For example, rodents are reservoir hosts for *B. afzelii* and *B. bavariensis*, whereas *B. valaisiana* and *B. garinii* use birds as reservoir hosts [1,16]. Many large animals are not only considered incompetent as hosts for spirochetes of the *B. burgdorferi* s.l. complex, but rather even zooprophylactic. Such observation was reported by Richter and Matuschka (2006) after testing questing *I. ricinus* ticks sampled in and outside a cattle pasture. The presence of cattle limited the prevalence of *B. burgdorferi* s.l. in ticks (3.7% of adult ticks and 5.8% of nymphs tested positive in pasture where cattle were present versus 40.3% of adults and 22.1% of nymphs positive from a site on which cattle did not graze). The zooprophylactic effect of these incompetent hosts becomes obvious when they compete with reservoir-competent animals as hosts for ticks, diverting as such the vectors from feeding on spirochetes reservoir-competent hosts [17]. However, they serve as important maintenance hosts for ticks by providing blood meals for all developmental stages [1,18], and by feeding female adults, they increase the availability of vectors.

Several studies reported the presence of *B. burgdorferi* genospecies in tick populations from Germany, indicating high infection rates (36.2%) in the southern part of the country [19,20,21,22,23,24,25,26,27,28,29]. The majority of these studies detected Lyme disease agents in questing ticks collected from woodlands and engorged ticks from hosts, and more work will be necessary to determine the differences of infection levels and genospecies diversity in ticks from different habitats and the potential influence of the feeding hosts on the abundance of *B. burgdorferi* s.l.

The aims of this study were therefore (1) to compare prevalence rates with *B. burgdorferi* s.l. in questing *I. ricinus* ticks from different habitat types, (2) to analyse species distribution of the *B. burgdorferi* s.l. complex according to habitat type, and (3) to analyse hosts feeding *I. ricinus* ticks for their infection rates with *B. burgdorferi* s.l.

## 2. Materials and Methods

### 2.1. Study Sites

The study sites were previously described in detail [30]. Altogether, six sites were included in the study, representing three different habitat types: urban parks (4 sites), pasture (1 site), and natural forest (1 site). All sites were located in the federal state of Bavaria, Germany. The higher number of urban sites included in this study is justified by the higher probability of ticks to feed on humans compared to pasture or natural habitats.

The urban sites are located in the cities of Munich (M2 and M3) and Regensburg (R1) and near Lake Starnberg in Munich (B). Sampling site M2 (‘English Garden’, 375 ha) is a city center park under a high anthropogenic influence, being frequently visited by people and dogs. Roe deer (*Capreolus capreolus)* and wild boar (*Sus scrofa*) are absent in this park, while mice, hedgehogs (*Erinaceus europaeus*), foxes (*Vulpes vulpes*), and birds are present. Sampling site M3 (‘Nymphenburger-Schlosspark’, 141 ha) has a higher density of trees, where wild animals such as roe deer are present, this city park being included in a nature conservation program.

Urban sampling site R1 (‘Dörnbergpark’, 7.4 ha) is a small park in the Regensburg inner city where large wild mammals such as wild boar or roe deer are absent, but 26 bird species have been described as common at this location.

Sampling site B (‘Schlosspark-Berg’, 30 ha) is a forested park with scrub and walkways, on the east side of Lake Starnberg. Wild animals such as roe deer and foxes are present at this site.

The pasture habitat type K (Kerschlach), represented by two fenced pastures (each of approx. 8 ha), is used to breed cattle, and it is surrounded by forest. Ticks were collected inside the fences.

The natural site (T, ‘Angelberger Forst’, 641 ha) is represented by a forest included in a nature conservation program where several wild animals occur but there is a low frequency of visitors.

### 2.2. Collection of Samples

Questing ticks were collected at all six sites in April, May, and June 2011 using the flagging method as previously described [30]. Wild boars (*Sus scrofa*) were sampled from October 2010 to February 2013, and roe deer (*Capreolus capreolus*) samples were collected from September 2010 to January 2012 at the natural site. All animals except one roadkill were professionally hunted, and the available samples were collected from animals harvested by regular hunting (during the regular hunting season). Altogether, 24 spleen, 21 blood, and 12 skin samples were collected from wild boar. Additionally, 16 engorged *I. ricinus* adult ticks were collected from 2 of the 24 wild boars [31]. From roe deer, altogether 95 spleen-, 86 blood-, and 56 skin-samples, as well as 557 adult *I. ricinus* ticks from 44 roe deer, were collected [30].

Furthermore, 64 adult engorged *I. ricinus* ticks were collected from 31 cattle on the pasture site.

### 2.3. DNA Extraction

DNA was isolated from all tissue samples and questing and engorged ticks with the automated Maxwell^®^ 16 System and the Maxwell^®^ 16 LEV Blood DNA Kit (Promega, Mannheim, Germany). Details of the DNA extraction have been published elsewhere [30].

### 2.4. PCR Screening

All samples were screened for *Borrelia burgdorferi* s.l. with a conventional PCR targeting the *rrfA-rrlB* (5S-23S rDNA) intergenic spacer (IGS) [32] using primers IgsA 5′-CGA CCT TCT TCG CCT TAA AGC-3′ and IgsB 5′-AGC TCT TAT TCG CTG ATG GTA-3′.

The Hot Star Taq Plus Kit (Qiagen, Hilden, Germany) was used in a 50 µL reaction volume in the Thermocycler Mastercycler^®^ gradient (Eppendorf, Hamburg, Germany) and at the following cycle conditions: initial activation at 94 °C for 5 min, followed by 39 cycles with denaturation at 94 °C 15 s, annealing at 55 °C 30 s, and extension at 72 °C 30 s. Final extension was 72 °C for 5 min. PCR-clean water as negative control and a positive control (DNA extracted from *Borrelia afzelii* culture) were included in every PCR run. PCR products were analysed by gel electrophoresis on a 2% agarose gel (Top Vision Agarose; Fermentas, St. Leon-Rot, Germany) dyed with Gel Red^TM^ Nucleid Acid stain (Biotium, Hayward, CA, USA). Visualization was performed under UV-light (PeqLab, Erlangen, Germany).

### 2.5. Species Identification by RFLP and Sequencing

**Figure 1:**
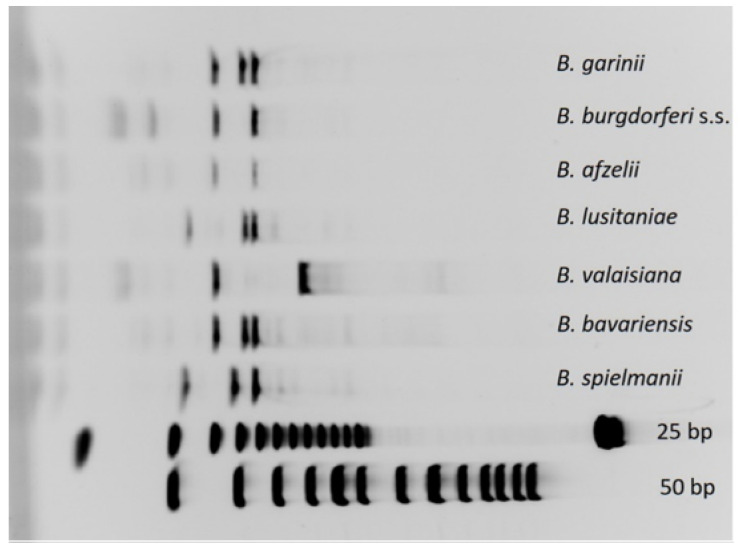
RFLP patterns of the defined *Borrelia* species used as positive controls for the species differentiation in tick samples.

Species identification of *Borrelia burgdorferi* s.l. was carried out with a restriction fragment length polymorphism (RFLP) protocol [32]. In brief, 13 µL of *B. burgdorferi* s.l. IGS positive products were transferred to a 1.5 mL tube (Eppendorf, Hamburg, Germany) and digested with 0.5 µL True 1I restriction enzyme (True 1I (MseI)-10 units/µL) and 1.5 µL buffer R (Fermentas, St. Leon-Rot, Germany) at 65 °C for 5–16 h with a mixing speed of 300 rpm. After adding 3 µL 6× DNA-Loading Dye (Fermentas, St. Leon-Rot, Germany), the samples were loaded on precast gels (Novex^®^ TBE Gels, 4–20%, 1.0 mm, 15 wells) running in a classical vertical electrophoresis (XCell SureLock^®^ Mini-Cell) (Life Technologies, Darmstadt, Germany). A 25 bp (Promega) and 50 bp DNA ladder (Fermentas, St. Leon-Rot, Germany) were used as standards. Samples were run in 1× TBE (Fermentas, St. Leon-Rot, Germany) at 200 V for 1 h. The gel was stained for 1 h with Gel Red^TM^ (Biotium, Eching, Germany), using a final Gel Red^TM^ concentration of 3× and UV visualized (PeqLab, Erlangen, Germany). Species identification was carried out by comparing the RFLP patterns with those of defined positive controls (in vitro cultured *Borrelia garinii*, *Borrelia burgdorferi* sensu stricto, *Borrelia afzelii*, *Borrelia lusitaniae*, *Borrelia valaisiana*, *Borrelia bavariensis,* and *Borrelia spielmanii*; each positive control was investigated for RFLP pattern (Figure 1) followed by sequencing). When species definition by RFLP patterns was not clear, PCR was repeated as described above, and positive PCR products were purified using the QIAquick PCR Purification Kit (Qiagen, Hilden, Germany) according to the manufacturer’s protocol and sequenced. Sequencing was performed by Eurofins MWG Operon (Ebersberg, Germany). Results were analyzed by Chromas Lite^®^. Reverse sequences were reversed and complemented with (http://www.bioinformatics.org/sms/rev_comp.html, accessed on 7 February 2013) and aligned with ClustalW2 (http://www.ebi.ac.uk/Tools/msa/clustalw2/, accessed on 7 February 2013). Database searches and sequence comparison were done with the BLAST tool provided by the National Center for Biotechnology Information (http://blast.ncbi.nlm.nih.gov/Blast.cgi, accessed on 6 February 2013) and with Geneious Prime 2021.0.1 (https://www.geneious.com accessed on 7 February 2013).

### 2.6. Statistical Analysis

Statistical differences were calculated with GraphPad Prism version 9.0.0 for Windows (GraphPad Software, La Jolla, CA, USA, www.graphpad.com). Ordinary one-way analysis of variance (ANOVA) and Tukey’s multiple comparison tests were performed on tick infection rates for different geographic groups, identified *Borrelia* species, and habitat categories. To analyse the differences in prevalence rates between tick developmental stages and between questing and feeding ticks, a Mann–Whitney test was performed. Differences were considered significant when *p* < 0.05.

## 3. Results

### 3.1. Questing Ticks

The collection of questing ticks resulted in a total of 2186 ticks, all *Ixodes ricinus* (580 females, 613 males, 760 nymphs, and 233 larvae). A total of 1482 ticks were collected from the urban habitat type: 454 females, 435 males, 500 nymphs, and 93 larvae. The number of ticks collected from each urban site is as follows: 362 ticks from site M2 (120 of each females, males, and nymphs, and two larvae), 401 ticks from site M3 (135 females, 124 males, 140 nymphs, and two larvae), 393 ticks from site R1 (120 of each females, males, and nymphs, and 33 larvae), and 326 ticks from site B (79 females, 71 males, 120 nymphs, and 56 larvae). From pasture, 366 *I. ricinus* ticks were obtained (93 females, 132 males, 140 nymphs, and one larvae), while 338 ticks were collected from the natural habitat type (33 females, 46 males, 120 nymphs, and 139 larvae).

### 3.2. Prevalence of Borrelia Burgdorferi Sensu Lato

Altogether, 469 out of 1953 questing ticks (24%) were positive for *Borrelia burgdorferi* s.l. (355 out of 1193 adult ticks (29.8%) and 114 out of 760 questing nymphs (15%)) (Table 1). All 233 larvae (54 pools, 1–5 ticks per pool) were negative. The prevalence at the natural site (13.6%) was lower than at the pasture site (28%) (*p* < 0.001) and at all urban sites (24.5%) (*p* < 0.01). Prevalence was lower at the urban sites with occurrence of roe deer (sites M3 and B) than on urban sites with absence of those wild ungulates (sites M2 and R1) (*p* < 0.01). Nymphs were less frequently infected (15%) than adult ticks (29.8%) (*p* < 0.001), while no statistical difference was observed between the infection rates of females (32.8%) and males (26.9%) (*p* = 0.14). *Borrelia burgdorferi* s.l. DNA was not detected in any of the blood and tissue samples of roe deer and wild boar from the natural site, but in 16/331 (4.8%) of engorged adult ticks from roe deer and in 1/16 (6.3%) from wild boar (Table 1). Additionally, 5 out of 64 (7.8%) engorged ticks collected from cattle from the pasture site were also positive.

When comparing *B. burgdorferi* s.l. infection rates between questing and engorged ticks, a significant difference is observed between the overall prevalence of questing ticks and the prevalence of engorged ticks from roe deer (24% vs. 4.8%; *p* < 0.001) and cattle (24% vs. 7.8%; *p* = 0.008). Additionally, questing females had also higher infection levels of *B. burgdorferi* s.l. compared to engorged females collected from roe deer (32.8% vs. 1.5%; *p* < 0.001).

Table 1 includes detailed data regarding the *B. burgdorferi* s.l. infection rates according to site, habitat type, and tick developmental stages.

### 3.3. Species Identification of the B. burgdorferi s.l. Complex

#### 3.3.1. Questing Ticks

**Figure 2:**
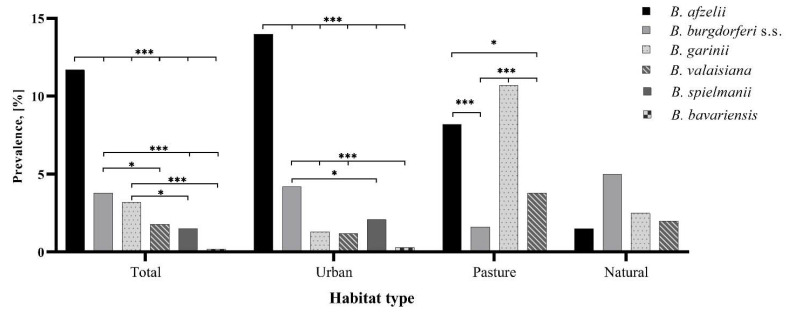
Overall prevalence of detected *Borrelia* genospecies in each habitat type. The percentages of positive ticks per genospecies for every single habitat are given. Significant differences between species infection rates are indicated above the bars; *, *p* < 0.05; ***, *p* < 0.001.

Based on the RFLP results, 218 samples (from a total of 469 *B. burgdorferi* s.l. positive questing ticks) were identified at species level, and the rest of 251 samples needed to be confirmed by sequencing, whereof 224 sequences were obtained. From the remaining 27 samples sequenced, 23 samples had unclear results, while for four sequences, there was no successful differentiation between *Borrelia garinii* and *Borrelia bavariensis* genospecies. In case of doubt, sequencing results were preferred to RFLP patterns for species identification. The identified *Borrelia* species had the following overall prevalence in ticks: *B. afzelii*, 11.7% (15% of adults and 6.4% of nymphs); *B. burgdorferi* s.s., 3.7% (4.8% of adults and 2.4% of nymphs); *B. garinii*, 3.2% (3.3% of adults and 3% of nymphs); *B. valaisiana*, 1.8% (2.4% of adults and 0.8% of nymphs); *B. spielmanii*, 1.5% (1.9% of adults and 0.8% of nymphs); and *B. bavariensis,* 0.2% (0.2% of adults and 0.3% of nymphs). The overall infection rates and the statistically significant differences between *Borrelia* genospecies in each habitat type are displayed in Figure 2. Out of the total sequences that matched *B. afzelii*, 63 had 99–100% and six had 95.6–98.7% similar identity to isolates from Finland (GenBank accession number: JX909859), France (KY273112), Estonia (KX418640), China (MK333414), Turkey (AB091798), or Russia (MK118756). Three *Borrelia burgdorferi* s.s. sequences had 97.3–98.9% similarity, while 43 had 99–100% similar identity to the sequences from Germany (Z77172), China (MK333419), and the USA (CP031412). Three isolates retrieved as *B. garinii* showed 95.9–98.7% similar identity, and 59 sequences were 99.1–100% similar to those from Spain (MG356956 and MK256778), France (CP028861 and KY27310), Russia (AB178358), and Sweden (JX909973). Two *Borrelia valaisiana* sequences from this study were 97.9–98.3% similar, and five sequences had 99.6–100% similarity to isolates from Czech Republic (AF497986), Russia (CP009117), and Turkey (AB091795). Five *Borrelia spielmanii* isolates had 96.4–98.4% similar identity, and 19 were 99–100% similar to isolates from Czech Republic (JX448322) and Germany (AM160605), while one *B. bavariensis* sequence showed 94.6% and other three had 99.1–100% similarity to one isolate from Germany (CP028872). In addition, seven isolates had 99–100% similar identity, while one sequence showed 97.8% similarity to uncultured *Borrelia* sp. from Sweden (HM173544) and Netherlands (MN515338).

When calculating the rates of identified species from the *B. burgdorferi* s.l. positive ticks, *B. afzelii* was identified in almost half of the positive samples (48.6%), being significantly different compared to *B. burgdorferi* s.s., *B. garinii, B. valaisiana, B. spielmanii,* and *B. bavariensis* (*p* < 0.001). The second most abundant genospecies, *B. burgdorferi* s.s. (16%), had a higher prevalence when compared to *B. valaisiana* (*p* = 0.007)*, B. spielmanii,* and *B. bavariensis* (*p* < 0.001), while no statistical difference was observed in comparison to *B. garinii* infection rate (*p* = 0.87). Table 2 contains detailed data for the rates registered by the *Borrelia* genospecies in positive ticks according to site and habitat type.

In relation to the habitat type, infection rate with *B. afzelii* of *B. burgdorferi* s.l. positive ticks from urban sites (57.4%) was significantly higher than of samples collected in the natural site and pasture (*p* < 0.001). No statistical difference was observed between the *B. afzelii* infection levels of ticks from the natural site and pasture (*p* = 0.15) (Table 2).

Ticks positive for *B. burgdorferi* s.s. from the natural site showed the highest prevalence (37.0%) when compared to the infection rates of ticks from urban sites (*p* = 0.019) and the pasture site (*p* < 0.001). Regarding the prevalence of positive ticks for *B. garinii*, samples from the pasture site showed significantly higher levels of infection compared to those from the urban sites (*p* < 0.001) and natural site (*p* = 0.005). *B. valaisiana* infected ticks from the natural site had the highest prevalence, but that was not significant when compared to the urban sites (*p* = 0.06) and pasture site (*p* = 0.91) (Table 2).

#### 3.3.2. Engorged Ticks (Cattle)

Five engorged tick samples from cattle were identified with the RFLP patterns, and three of them needed to be confirmed by sequencing. Altogether 2/5 (40%; two females) positive samples were 99.5–100% identical to *B. garinii* (GenBank accession nos. MK256778 and CP028861), 2/5 (40%) samples were identified as *B. afzelii* (one female positive after RFLP and one male showing 99.5% similarity to GenBank accession no. KY273111), and 1/5 (20%; one female) positive sample identical to *B. valaisiana* (only RFLP, no sequencing result) (Table 2).

#### 3.3.3. Engorged Ticks (Roe Deer)

After analysing the RFLP patterns, 16 engorged tick samples from roe deer tested positive for *B. burgdorferi* s.l. DNA, and sequencing returned valid sequences for 14 samples. Altogether, 7/16 (43.8%; two females, five males) of the positive tested samples were 98.6–100% identical to *B. garinii* (GenBank accession nos. MG356949, AB178358, and CP028861), 4/16 (25.0%; one female, three males) samples were 99.5–100% similar to *B. afzelii* (GenBank accession nos. AY772046, KX418638, MK333414, and KY273113), 2/16 (12.5%; two males) positive samples showed 97.3 and 98% similar identity to *B. burgdorferi* s.s. (GenBank accession nos. MK333419 and CP031412), and one sequence matched 100% to *Borrelia* sp. (AF090983) (Table 2).

#### 3.3.4. Engorged Ticks (Wild Boar)

The one positive tick sample after RFLP (one female) was confirmed by sequencing, and the resulting sequence showed 100% similar identity to *B. burgdorferi* s.s. (GenBank accession no. MK333419).

Sequences obtained in this study from both questing and engorged ticks were deposited in GenBank under the following accession nos.: MW489011-MW489234 and MW545809-MW545823.

## 4. Discussion

Compared to other available studies from Germany, the prevalence of 29.8% of *B. burgdorferi* s.l. in questing adult ticks in the present study ranges at the upper level of reported infection rates [24,29,33,34,35,36,37]. In addition, *I. ricinus* nymphs showed high infection rates as well (15%), indicating that the selected study areas represent high-risk zones for acquisition of *Borrelia*. The difference in infection rates between adult ticks and nymphs found in our study is consistent with data reported by several previous studies; the existing difference may be explained by the fact that two blood meals are required in order to reach the host-seeking adult stage; hence, greater chances exist to acquire the spirochetes [38]. Nymphs also pose a risk to public health due to the small size, high abundance, and frequency of feeding on human hosts while remaining virtually undetected [39].

We observed that the samples collected from the natural habitat had significantly lower *B. burgdorferi* s.l. infection rates when compared to those from urban sites and pasture. These results somehow contravene the current knowledge stating that *B. burgdorferi* s.l. is more abundant in the forested natural habitats [40]. However, the urban sites and pasture from this study are situated within or in close vicinity to forested areas, and several domestic and wild animals have been observed in these habitats [30]. The existence of suitable conditions for maintaining the tick populations and the presence of competent reservoir hosts for *Borrelia* genospecies are the main characteristics that can explain the high prevalence of *B. burgdorferi* s.l in ticks from these sites. High infection rates in questing *I. ricinus* from urban and peri-urban locations have been reported: in France (20.6% of questing ticks collected from a suburban forest) [41], Hamburg, Germany (34.1% of ticks flagged from recreational areas positive for *B. burgdorferi* s.l.) [21], and Hanover, Germany (24% of ticks positive for *Borrelia* species) [24].

Although roe deer in our study were infested with *B. burgdorferi* s.l. positive ticks, *Borrelia* DNA was not detectable in any animal. Roe deer seem to serve as a blood source for ticks without having reservoir competence for *Borrelia* spp. [42]. When comparing the prevalence of *B. burgdorferi* s.l. in questing ticks from the natural habitat (21.5% of adult ticks) to engorged ticks collected from roe deer (4.8% of adult ticks), it is clear that diverting ticks to feed on incompetent reservoir hosts such as roe deer has as an outcome the reduction of positive ticks. *B. burgdorferi* s.l. infection levels in questing ticks from urban sites also registered a lower prevalence at sites with occurrence of roe deer (sites M3 and B) than at the sites where these wild ungulates were absent (sites M2 and R1).

In addition to the contribution of ticks failing to acquire the pathogen, feeding of infected ticks on roe deer and other wild ungulates causes the loss of infection, probably as a result of the bacteriolytic activity of the complement pathway [43]. This has been reported by other studies as well [17,44,45]. The results of our study support this theory, especially by taking a closer look at the infected *I. ricinus* females from the natural habitat. In this case, the difference in prevalence between *B. burgdorferi* s.l. positive questing females (27.3%) and positive engorged females (1.5%) collected from their roe deer hosts is even greater.

The methods used in the current study to determine *B. burgdorferi* s.l. genospecies (RFLP and PCR) allowed the identification of six different species. *Borrelia afzelii* showed the highest rate of infection in positive questing ticks, which corresponds to the published reports from Europe. This species is also being recognised as the most common cause of Lyme disease in Europe [8]. The high prevalence of *B. afzelii* in ticks is believed to be the result of the abundance and spread of rodents, the main reservoir hosts [1,16,38], but may vary between habitats [10].

When compared between habitat types, questing ticks from urban sites tested positive for all six genospecies found in this study, while in ticks from natural and pasture sites, only four genospecies were detected. These results could suggest the suitable conditions for *Borrelia* genospecies to occur in urban habitats compared to other habitat types.

*Borrelia afzelii* had the highest prevalence in ticks from urban sites, especially in those where the occurrence of small mammals and the absence of large mammals have been documented (sites M2 and R1), facilitating the maintenance of this genospecies [30]. The *B. afzelii* isolates from this study matched with sequences from different countries from Europe and Asia and considering the short fragment amplified for *Borrelia* species determination (~230 to 250 bp), more in-depth analysis such as multi-locus sequence typing (MLST) would be required for phylogenetic and evolutionary relationship studies. The second most common genospecies was *B. burgdorferi* s.s., followed by *B. garinii*. Both these genospecies are pathogenic to humans [1] and are commonly encountered in Central and Western Europe [3,46]. Small mammals and birds are the reservoir hosts for *B. burgdorferi* s.s., while only birds seem to host *B. garinii* [47,48,49]. With regard to habitat type, *B. burgdorferi* s.s. infection rates in questing ticks contrasted with the general prevalence for *B. burgdorferi* s.l. or for *B. afzelii* and *B. garinii*, high infection levels were observed in ticks from urban sites with the presence of large mammals (M3 and B) and from natural habitat. While the high prevalence in the natural habitat may be explained by the abundance of vector ticks and competent reservoir hosts, there is no convincing explanation for the higher prevalence at urban sites of the occurrence of roe deer. It could be that the high abundance of *B. burgdorferi* s.s. is favored by deer as well, by keeping the tick population high, hence the higher risk for humans of getting infected with *Borrelia*. Similar data have been reported from the USA where white-tail deer densities and *Ixodes* ticks abundance correlated positively with human cases of Lyme disease [50], even though it was shown that deer have a zooprophylatic effect on *B. burgdorferi* s.s. in ticks [51].

In addition to the already-mentioned pathogens, RFLP and PCR from questing tick samples successfully identified other known human pathogenic genospecies: *Borrelia spielmanii* (4.7%) and *Borrelia bavariensis* (0.9%) and the non-human pathogenic species *B. valaisiana* (7.2%). Birds are the competent reservoir hosts for *B. valaisiana*, whereas small mammals are hosts for *B. spielmanii* and *B. bavariensis* [26]. *Borrelia valaisiana* was detected in questing ticks from all habitat types and collection sites, suggesting that immature stages of ticks commonly feed on birds, enhancing a broad distribution of the species in tick populations. As for *B. spielmanii* and *B. bavariensis*, positive ticks were detected only in urban sites and at higher rates at the sites with no occurrence of roe deer. This can indicate a potential increase in the prevalence for these two pathogens due to the lack of dilution effect for which incompetent reservoir hosts and the host species-rich communities are responsible [15].

## 5. Conclusions

The current study increases the information regarding *Ixodes* infection by *B. burgdorferi* s.l. in different habitats from Southern Germany, describes the diversity of genospecies in questing and engorged ticks from the selected sites, and shows a comparison between infection levels of ticks from different types of habitats. The main findings refer to the overall high infection levels and the diversity of *Borrelia* genospecies in questing ticks from urban sites, pasture, and natural habitats. The low *B. burgdorferi* s.l. infection levels in ticks from sites where roe deer were present suggest a zooprophylactic effect that these wild mammals might have against this pathogen.

## Figures and Tables

**Table 1:** Prevalence of *Borrelia burgdorferi* sensu lato in questing and engorged *Ixodes ricinus* ticks (collected from roe deer, wild boar, and cattle) per gender, stage, site, and habitat.

*Feeding Status*	Habitat	Site	Female			Male			Nymph			Total		
			Pos/No. Total	%	95% CI	Pos/No. Total	%	95% CI	Pos/No. Total	%	95% CI	Pos/No. Total	%	95% CI
Questing ticks	Urban	M2	46/120	38.3	29.6–47.7	47/120	39.2	30.4–48.5	19/120	15.8	9.8–23.6	112/360	31.1	26.5–36.1
		M3	28/135	20.7	14.3–28.6	30/124	24.2	17.0–32.7	22/140	15.7	10.1–22.8	80/399	20.1	16.4–24.3
		R1	45/120	37.5	28.8–46.8	32/120	26.7	19.0–35.5	29/120	24.2	16.8–32.8	106/360	29.4	24.9–34.4
		B	29/79	36.7	26.1–48.3	13/71	18.3	10.1–29.3	0/120	0.0	0.0–3.0	42/270	15.6	11.7–20.4
		Total	148/454	32.6	28.3–37.1	122/435	28.0	23.9–32.5	70/500	14.0	11.1–17.4	340/1389	24.5	22.3–26.8
	Pasture	K	33/93	35.5	25.8–46.1	35/132	26.5	19.2–34.9	34/140	24.3	17.4–32.3	102/365	27.9	23.6–32.8
	Natural	T	9/33	27.3	13.3–45.5	8/46	17.4	7.8–31.4	10/120	8.3	4.1–14.8	27/199	13.6	9.5–19.0
	All sites	Total	190/580	32.8	29.0–36.7	165/613	26.9	23.4–30.6	114/760	15.0	12.5–17.7	469/1953	24.0	22.2–26.0
Cattle	Pasture	K	4/60	6.7		1/4	25		na ^(1)^	na ^(1)^	na ^(1)^	na ^(1)^	na ^(1)^	na ^(1)^
Roe deer	Natural	T	3/206	1.5		13/125	10.4		na ^(1)^	na ^(1)^	na ^(1)^	na ^(1)^	na ^(1)^	na ^(1)^
Wild boar			1/15	6.7		0/1	0.0		na ^(1)^	na ^(1)^	na ^(1)^	na ^(1)^	na ^(1)^	na ^(1)^

M2 = urban site ‘English Garden’; M3 = urban site ‘Nymphenburger-Schlosspark’; R1 = urban site ‘Dörnbergpark’; B = urban site ‘Schlosspark-Berg’; K = pasture site ‘Kerschlach’; T = natural site ‘Angelberger Forst’; ^(1)^ na = not applicable.

**Table 2:** *Borrelia burgdorferi* sensu lato species in questing and engorged *Ixodes ricinus* ticks per site and habitat.

*Ixodes ricinus*	Habitat	Site	Total Pos	*B. afzelii* (*n*/%)	*B. burgdorferi* Sensu Stricto (*n*/%)	*B. garinii* (*n*/%)	*B. valaisiana* (*n*/%)	*B. spielmanii* (*n*/%)	Uncultured *Borrelia* (*n*/%)	*B. bavariensis* (*n*/%)	*Borrelia* sp. (*n*/%)	*B. garinii/B. bavariensis* (*n*/%)	Unknown (*n*/%)
Questing ticks	Urban	M2	112	71/63.4	13/11.6	13/11.6	5/4.5	2/1.8	0	2/1.8	0	0	6/5.4
		M3	80	37/46.3	25/31.3	4/5.0	1/1.3	5/6.3	6/7.5	0	0	0	2/2.5
		R1	106	66/62.3	6/5.7	0	7/6.6	22/20.8	0	2/1.9	0	0	3/2.8
		B	42	21/50.0	15/35.7	1/2.4	4/9.5	0	0	0	0	0	1/2.4
		Total	340	195/57.4	59/17.4	18/5.3	17/5.0	29/8.5	6/1.8	4/1.2	0	0	12/3.5
	Pasture	K	102	30/29.4	6/5.9	39/38.2	14/13.7	0	1/1.0	0	0	4/3.9	8/7.8
	Natural	T	27	3/11.1	10/37.0	5/18.5	4/14.8	0	1/3.7	0	1/3.7	0	3/11.1
	All sites	Total	469	228/48.6	75/16.0	62/13.2	35/7.5	29/6.2	8/1.7	4/0.9	1/0.2	4/0.9	23/4.9
Cattle	Pasture	K	5	2/40.0	0	2/40.0	1/20.0	0	0	0	0	0	0
Roe deer	Natural	T	16	4/25.0	2/12.5	7/43.8	0	0	0	0	1/6.3	0	2/12.5
Wild boar			1	0	1/100	0	0	0	0	0	0	0	0

M2 = urban site ‘English Garden’; M3 = urban site ‘Nymphenburger-Schlosspark’; R1 = urban site ‘Dörnbergpark’; B = urban site ‘Schlosspark-Berg’; K = pasture site ‘Kerschlach’; T = natural site ‘Angelberger Forst’; *n* = number of positive samples.

## Data Availability

Sequences obtained in this study were deposited in GenBank (http://www.ncbi.nlm.nih.gov/genbank/) under the following accession nos.: MW489011-MW489234 and MW545809-MW545823.
